# Carbon Fiber Paper Sensor for Determination of Trimethoprim Antibiotic in Fish Samples

**DOI:** 10.3390/s23073560

**Published:** 2023-03-29

**Authors:** Álvaro Torrinha, Miguel Tavares, Vitória Dibo, Cristina Delerue-Matos, Simone Morais

**Affiliations:** REQUIMTE-LAQV, Instituto Superior de Engenharia do Porto, Instituto Politécnico do Porto, Rua Dr. António Bernardino de Almeida, 431, 4249-015 Porto, Portugal

**Keywords:** electrochemical sensor, carbon paper, trimethoprim, electroanalysis, fish, environmental analysis

## Abstract

The increase in anthropogenic pollution raises serious concerns regarding contamination of water bodies and aquatic species with potential implications on human health. Pharmaceutical compounds are a type of contaminants of emerging concern that are increasingly consumed and, thus, being frequently found in the aquatic environment. In this sense, an electrochemical sensor based on an unmodified and untreated carbon fiber paper (CPS—carbon paper sensor) was simply employed for the analysis of trimethoprim antibiotic in fish samples. First, the analytical conditions were thoroughly optimized in order for the CPS to achieve maximum performance in trimethoprim determination. Therefore, an electrolyte (0.1 M Britton–Robinson buffer) pH of 7 was selected and for square wave voltammetry parameters, optimum values of amplitude, frequency and step potential corresponded to 0.02 V, 50 Hz, and 0.015 V, respectively, whereas the deposition of analyte occurred at +0.7 V for 60 s. In these optimum conditions, the obtained liner range (0.05 to 2 µM), sensitivity (48.8 µA µM^−1^ cm^−2^), and LOD (0.065 µM) competes favorably with the commonly used GCE-based sensors or BDD electrodes that employ nanostructuration or are more expensive. The CPS was then applied for trimethoprim determination in fish samples after employing a solid phase extraction procedure based on QuEChERS salts, resulting in recoveries of 105.9 ± 1.8% by the standard addition method.

## 1. Introduction

The increased unsustainability of fish resources is a reality caused by the overfishing [1] that is pressured by the food demand of an ever-growing world population [2]. In parallel, the increasing presence of pollutants from anthropogenic activities is also expected, negatively affecting aquatic ecosystems since, globally, 80% of wastewaters are released without any treatment [3]. Therefore, consumption of contaminated seafood is probable due to the bioavailability properties of many pollutants, which makes the adoption of preventive measures to improve food safety and reduce food wastes imperative.

Pharmaceutical compounds are an important class of contaminants of emerging concern given the vast number of different compounds available, their wide and increasing consumption, and their potential persistent, bioavailable, and toxic nature [4,5]. Even if properly discharged, the wastewater treatments may not be sufficient to guarantee their removal or degradation [6,7], which makes pharmaceutical compounds ubiquitous in the environment, as suggested by many studies [6,7,8,9,10,11,12]. Antibiotics are one of the most relevant groups of pharmaceutical compounds, and some have been found at high concentrations in waters [11,13]. In this regard, trimethoprim is a synthetic antibiotic, and one of the most consumed drugs [14]. The lack of information and regulation on the presence of this compound in the environment led to its inclusion on the European Commission’s surface water watch list [15]. Electrochemical methods, in particular voltammetry, provide ease of operation, high sensitivity, compatibility with miniaturization, and portability of the equipment, which can be further improved with the use of the advanced technology of sensors [16,17]. Therefore, sensor technology can become a very pertinent component in the analysis of single compounds such as trimethoprim, and contaminants in general [18,19,20].

In this work, an electrochemical sensor was developed for quantitative analysis of trimethoprim in fish samples. Carbon fiber paper was selected as transducing material without any pre-treatment and modification and used as a sensor (CPS—carbon paper sensor), being thoroughly optimized in order to achieve maximum analytical performance in fish matrices. This type of material has been emerging as a transducer in electrochemical sensors, due to its inherent mechanical, electrical, and electrochemical properties. In particular, carbon paper is a highly porous material composed of randomly arranged carbon fibers with micrometer diameter that translate to a high specific surface area and, thus, high sensitivities. Moreover, this material is lightweight and thin, resembling paper, which permits size and shape adjustments. These are interesting features when compared with other commonly employed electrodes in sensor fabrication, as evidenced in a recent review [21]. Concerning trimethoprim, some sensors have been developed based on more traditional electrodes such as glassy carbon (GCE) [22,23,24,25,26,27,28] or boron-doped diamond (BDD) electrode [29,30,31], but also screen-printed [32,33] and carbon paste [34] were employed (Table 1). Considering all these sensors, none were employed in challenging solid food samples, such as fish, which was tested for the first time in this study.

## 2. Materials and Methods

### 2.1. Materials, Reagents, and Solutions

All analytical grade chemicals were obtained and used without further purification.

Reagents such as acetaminophen, acetylsalicylic acid, sodium nitrate, trimethoprim, amoxicillin, sulfamethoxazole, and dimethyl sulfoxide were acquired from Sigma-Aldrich (Steinhein, Germany), while L-glutamic acid, calcium carbonate, sulfuric acid (97%), and hydrochloric acid (37%) were acquired from Fluka (Buchs, Switzerland). D(+)-glucose anhydrous was acquired from Scharlab (Sentmenat, Spain), whereas L(+)-ascorbic acid, D(+)-lactose monohydrate, and methanol were acquired from Riedel-de-Haën (Seelze, Germany). Ortho-phosphoric acid and sodium sulphate were obtained from Merck (Steinhein, Germany) and ortho-boric acid and potassium chloride (99.8%) from VWR (Leuven, Belgium). Glacial acetic acid, ethanol absolute anhydrous, and acetonitrile were purchased from Carlo Erba Reagents (Val-de-Reuil, France), whereas sodium hydroxide was obtained from Labkem (Barcelona, Spain).

Carbon paper (Toray TGP-H-60) was purchased from Alfa Aesar (Kandel, Germany). QuECHERS Classic (4 g magnesium sulfate and 1 g sodium chloride) was purchased from Teknokroma (Barcelona, Spain), whereas the dispersive SPE kit for drug residues in meat (150 mg C18 and 900 mg magnesium sulfate) was obtained from Agilent (Santa Clara, CA, USA).

Aqueous solutions were prepared using ultrapure water obtained from a Miliporewater purification system (18 MΩ, Milli-Q, Millipore, Molsheim, France). Britton–Robinson buffer (BRB) with 0.1 M concentration was used as the main electrolyte solution and prepared using sodium hydroxide, acetic acid glacial, phosphoric acid, boric acid, and potassium chloride. The pH was adjusted using 1 M NaOH or HCl. Stock solution of trimethoprim were prepared in methanol and then diluted with BRB when necessary.

### 2.2. Instrumentation and Electrochemical Measurements

**Figure 1:**
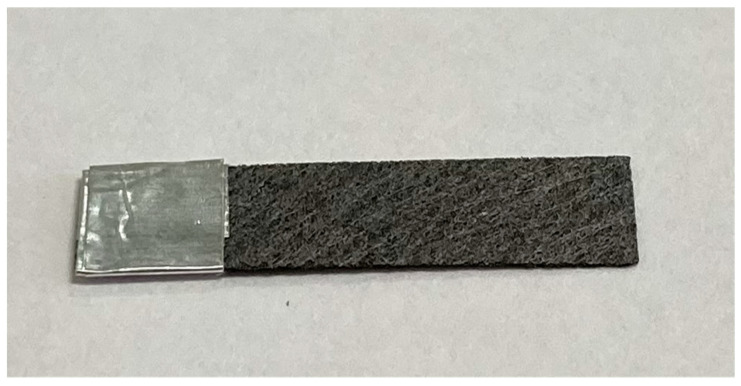
Homemade CPS with dimensions of about 2.5 × 0.7 cm^2^ containing aluminum foil at one end for better connection.

All electrochemical experiments were performed with a Metrohm potentiostat, model Autolab PGSTAT12, controlled by GPES v4.9 software (Herisau, Switzerland). The electrochemical characterization was carried on by cyclic voltammetry (CV) and square wave voltammetry (SWV) techniques in a three-electrode cell format composed of a Ag/AgCl (KCl, 3 M) reference electrode, a platinum counter electrode, and the CPS as working electrode (Figure 1). The CPS was simply assembled by cutting a rectangular piece with dimensions of about 2.5 × 0.7 cm^2^ (0.19 mm thickness) and covering one of the ends with aluminum foil for better connection with a crocodile clip. It was employed without any pre-treatment and modification of the surface. The current densities were obtained by dividing the peak current by the geometric area (about 0.63 cm^2^) of the CPS immersed in the electrolyte. Morphological characterizations of the untreated and pre-treated CPS were previously performed [38].

A series of optimizations of the analytical conditions were performed using SWV, namely, electrolyte pH (from 3 to 12), the technique parameters (amplitude, frequency, and step potential), and trimethoprim deposition (deposition time and potential). The calibrations curves were performed by SWV in the optimized conditions, with the electrolyte solution being stirred 15 s after each addition of trimethoprim stock solution for homogenization.

### 2.3. Real Sample Preparation and Analysis

The validation of the sensor was performed in fish samples of *Merluccius capensis* bought in a local supermarket (Porto, Portugal). About 1 g of edible meat (previously spiked with trimethoprim since no signal was obtained without fortification) was weighted (Thermo Fisher Scientific, model FPRS223, Leicestershire, UK) into a 50 mL falcon tube and then 5 mL of water and 5 mL of acetonitrile were added with the mixture being vortexed (VWR, VV3, UK) for 1 min. The QuEChERS salts (4 g magnesium sulfate and 1 g sodium chloride) were then added to the falcon tube and thoroughly shaken by hand for 1 min. Next, the mixture was centrifuged (Thermo Fisher Scientific Heraeus Megafuge 16R, Kandel, Germany) at 4000 rpm, 4 °C for 5 min, with the supernatant being collected and transferred to the dispersive solid-phase extraction (SPE) falcon tube. After being vortexed for 1 min, the mixture was again centrifuged at 13,000 rpm, 4 °C for 3 min. The final supernatant was collected and evaporated under a nitrogen (99.99%) stream. The residue was then redissolved in 1 mL of 30:70 acetonitrile:buffer (*v*/*v*), being finally analyzed through the standard addition method.

## 3. Results and Discussion

### 3.1. Electrochemical Behavior of Trimethoprim

**Figure 2:**
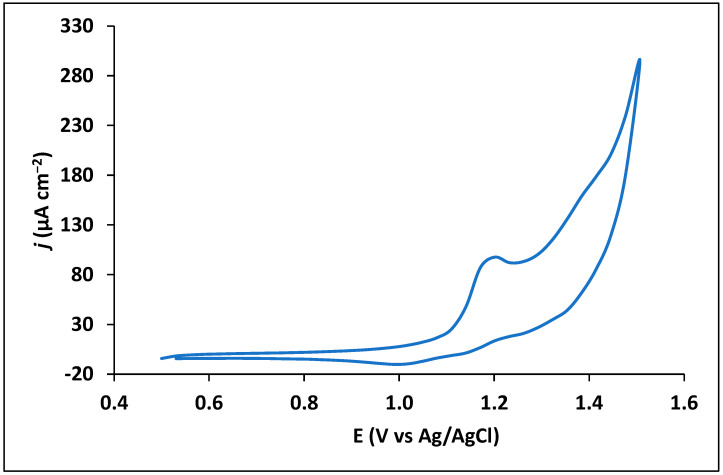
Cyclic voltammogram of 50 µM trimethoprim at 100 mV s^−1^. First scan.

Trimethoprim is an antibiotic drug that contains amino and oxygen functionalities in its chemical structure, being this way susceptible to electrochemical reaction processes. Preliminary experimental studies were performed in order to understand the electrochemical behaviour of trimethoprim drug using an unmodified CPS. A simple CV measurement showed a peak at around +1.2 V, indicating oxidation of the drug (Figure 2) with irreversible nature, in accordance with other studies [25,35,39].

**Figure 3:**
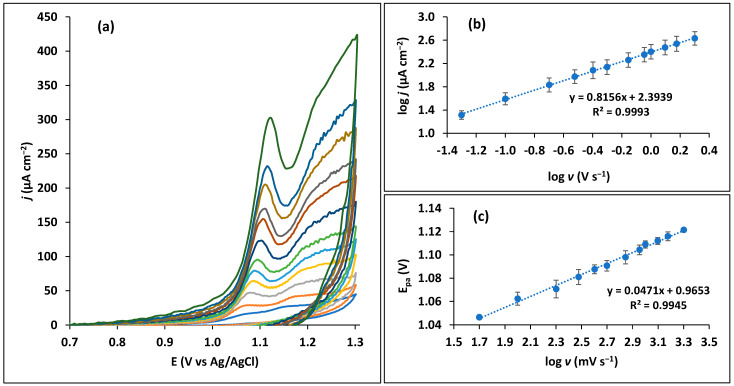
Scan rate study of 10 µM trimethoprim in 0.1 M Britton–Robinson buffer pH 7 on the carbon paper sensor. (**a**) Cyclic voltammograms at various scan rates (from 50 to 2000 mV s^−^^1^). (**b**) Logarithm of peak current as a function of logarithm of scan rate. (**c**) Peak potential as a function of the logarithm of scan rate.

Two different, but sensitive techniques were equated and compared (Figure S1) for this sensor, namely, differential pulse voltammetry (DPV) and square wave voltammetry (SWV). Both techniques seem suitable for the determination of trimethoprim, though for a low concentration corresponding to 0.1 µM, the SWV peak seemed more resolved compared with DPV, thus, being selected for subsequent studies. For higher concentrations, a second peak at more positive potentials can be observed despite being more faded than the first peak (Figure S1a,b). These two oxidation peaks, separated by about 0.1 V are likely attributed to the oxidation of both amino groups contained in the trimethoprim structure as suggested by Goyal and Kumar [39]. The peak at +1.1 V has more expression and, thus, is the one considered in this study. The type of electron transfer process between the CPS and trimethoprim was assessed by CV at different scan rates (50 to 2000 mV s^−1^), as depicted in Figure 3a. The plot of the logarithm of peak current as a function of the logarithm of scan rate dictates a linear relationship with a slope of 0.816 (*n* = 3) (Figure 3b). This value is between 0.5 and 1, which indicates a mixed electron transfer mechanism controlled by both diffusion and adsorption [40]. Other studies in the literature using different electrodes demonstrate that the process of trimethoprim oxidation can occur either controlled by diffusion [34], adsorption [22,28], or a combination of these two processes [25,26]. The number of electrons (*z*) involved in the oxidation reaction was assessed by applying the Laviron equation (Equation (1)) [41]:

(1) $$E_{pa}=E_{0}+\left(\frac{2.303RT}{\left(1-\alpha \right)zF}\right)\log\left(\frac{RTk^{0}}{\left(1-\alpha \right)zF}\right)+\left(\frac{2.303RT}{\left(1-\alpha \right)zF}\right)\log v$$

Plotting the peak potential at the different scan rates versus the logarithm of the scan rate, linearity is observed (Figure 3c), the slope being equal to 2.303*RT*/(1 − *α*)*zF*. Taking in consideration a transfer coefficient (*α*) of about 0.5 for this molecule [22,42], the calculated number of electrons, *z*, corresponded to 2.

### 3.2. Optimization of Analytical Conditions

**Figure 4:**
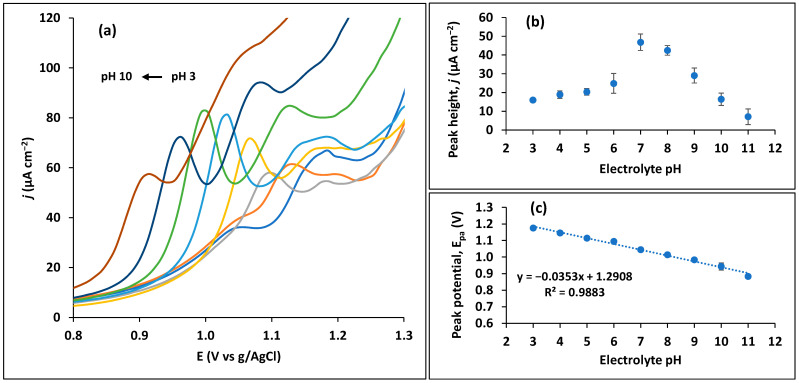
Optimization of electrolyte (0.1 M Britton–Robinson buffer) pH for 10 µM trimethoprim. (**a**) SWV curves from pH 3 to pH 12. (**b**) Influence of pH in trimethoprim peak height. (**c**) Influence of pH on trimethoprim peak potential.

**Figure 5:**
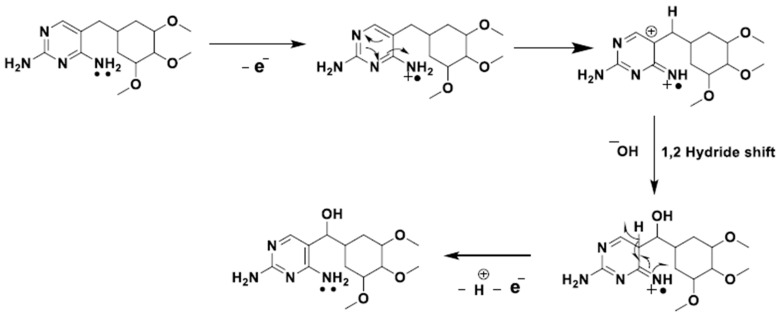
Possible two-electron oxidation mechanism of trimethoprim. (Reproduced from Patil et al. [42], with permission from MDPI, 2022).

The analytical conditions were optimized in order to enhance trimethoprim signal by the CPS. Starting with pH optimization, its effect on the oxidation peak height was measured by varying the electrolyte (0.1 M BRB) pH from 3 to 12 (Figure 4a). In all pH values, the SWV analysis of trimethoprim results in two oxidation peaks that are well-separated, however, only the first one is considered for analytical purposes based on the significantly higher peak current, as mentioned before. This way, a maximum peak around pH 7 is clearly seen in Figure 4b, being selected as the optimum value. This optimum pH is near the pK_a_ (6.6) [37] of trimethoprim, which means that it exists in ionized and nonionized forms in similar proportions in the solution [43]. This value is in line with the one found by Guaraldo et al. [26], though other studies identify optimum values of 3 [24,34,35] to 5 [22]. The plot between *E_pa_* and pH (Figure 4c) presents a shift of peak potential towards more negative values with increasing pH, suggesting the involvement of protons [39] in accordance with Equation (2) [44,45]:

(2) $$E_{p}=E^{0\prime }-\left(\frac{0.0592m}{z}\right)\mathrm{pH}$$

where *m* and *z* represent the number of mol of protons and electrons, respectively. The observed linear relationship presents a slope of 35.3 mV/pH, which indicates a different number of electrons and protons involved in the oxidation rection. Taking in consideration a two-electron transfer process, determined in Equation (1), the number of calculated protons corresponds to 1.2 (≈1), accordingly to Equation (2). Based on a study of the literature, the possible oxidation reaction of trimethoprim is depicted in Figure 5 [42].

**Figure 6:**
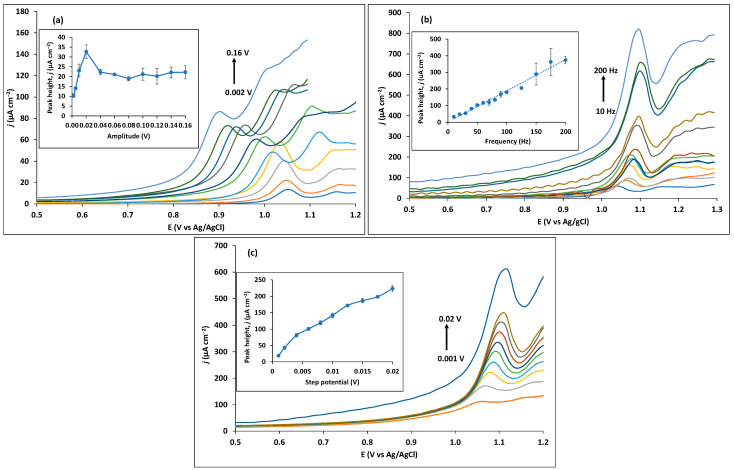
Optimization of SWV parameters for 10 µM trimethoprim in 0.1 M Britton–Robinson buffer pH 7. (**a**) Optimization of amplitude from 0.002 to 0.16 V. Inset: Peak height as a function of amplitude. (**b**) Optimization of frequency from 10 to 200 Hz. Inset: Peak height as a function of frequency. (**c**) Optimization of step potential from 0.001 to 0.02 V. Inset: Peak height as a function of step potential.

The SWV technique parameters may also significantly influence the analytical signal. This way, amplitude, step potential, and frequency were each individually optimized, while keeping the other parameters constant. When varying the amplitude from 0.002 to 0.16 V, it is possible to observe in Figure 6a that peak height raises sharply until 0.02 V, which then decreases and stabilizes. Therefore, an amplitude value of 0.02 V was selected as optimum and used in the following optimizations. Next, frequency was varied from 10 to 200 Hz (Figure 6b). Although peak height increases almost linearly, an unreproducible behaviour is noted for high values of frequency. The step potential varies from 0.001 to 0.02 V, which translates to a scan rate range between 100 and 2000 mV s^−1^ (Figure 6c). The peak height of trimethoprim constantly increases; however, here too, an unreproducible peak shape and significant noise with increasing scan rate is observed. To achieve a compromise between reproducibility and good signal, a frequency of 50 Hz and step potential of 0.015 V are chosen, which corresponds to a scan rate of 750 mV s^−1^.

**Figure 7:**
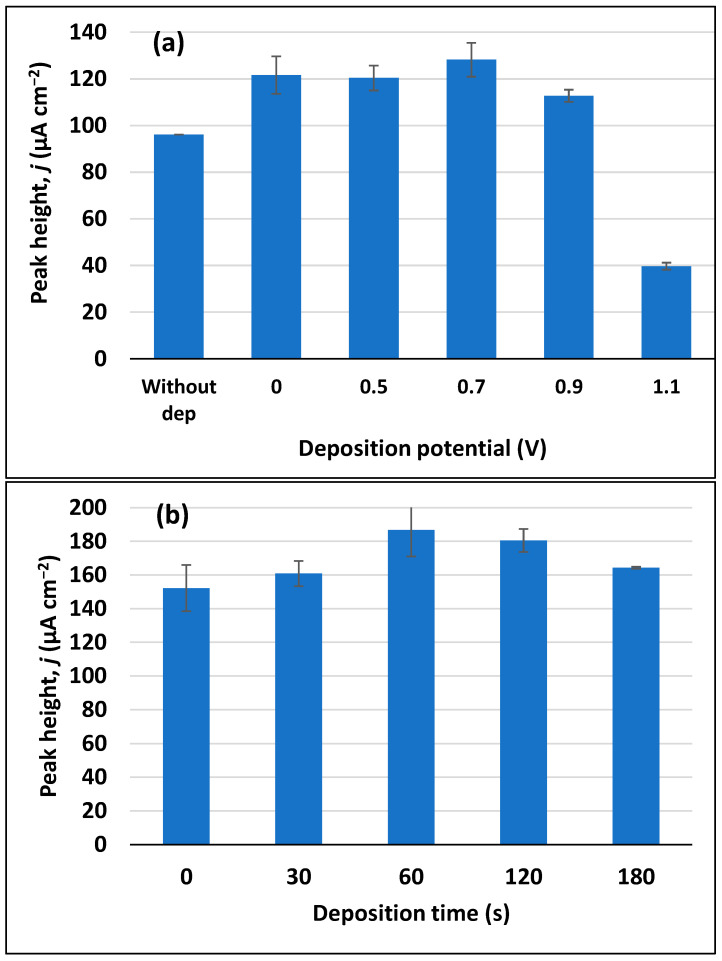
Optimization of deposition conditions for 10 µM trimethoprim in 0.1 M Britton–Robinson buffer pH 7. (**a**) Influence of deposition potentials on trimethoprim peak height (deposition time of 60 s). (**b**) Influence of deposition time on trimethoprim peak height.

Finally, electrodeposition of the analyte at the electrode surface was also assessed. Deposition potential was first optimized by applying different potentials for 60 s (Figure 7a). The peak height slightly increases for an applied potential of +0.7 V. Applying deposition potentials near the potential peak leads to the opposite effect, since it starts to oxidize trimethoprim before the start of the SWV measurement, which consequently lowers the obtained signal, as expected. The deposition times corresponding to 30, 90, 120, and 180 s were then tested, as depicted in Figure 7b, with 60 s being chosen as the optimum deposition time.

### 3.3. Analysis of Trimethoprim

**Figure 8:**
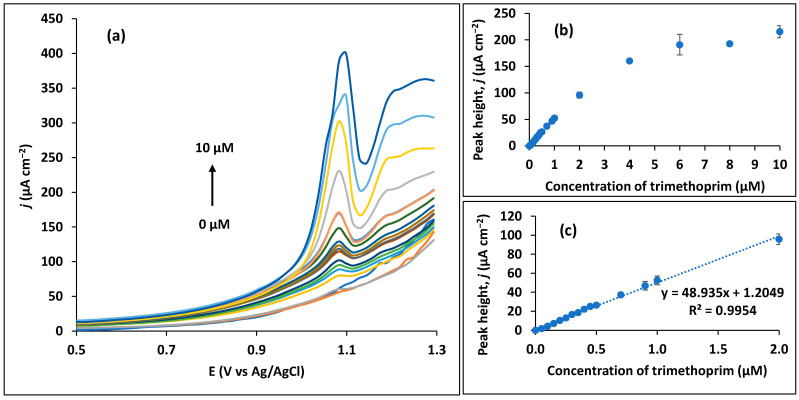
Assays for determination of the linearity range of trimethoprim in the optimum conditions (pH 7; SWV parameters: 0.02 V amplitude, 50 Hz frequency, and 0.015 V step potential; deposition of +0.7 V and deposition time of 60 s). (**a**) SWV curves up to 10 µM. (**b**) Peak height for all concentrations range tested (up to 10 µM). (**c**) Linear calibration range.

The analysis of trimethoprim was performed by SWV in the optimum conditions that were previously determined (BR buffer at pH 7; SWV parameters: 0.02 V amplitude, 50 Hz frequency, and 0.015 V step potential; deposition potential of +0.7 V and deposition time of 60 s). The calibration data (Figure 8) were obtained through consecutive standard additions of trimethoprim to the electrolyte solution. The wider calibration curve depicted in Figure 8b indicates a saturation and stabilization of peak height of trimethoprim for concentrations above 2 µM. Therefore, a good linearity is obtained between concentrations of 0.05 and 2 µM (Figure 8c). In this range, the mean sensitivity, which is retrieved from the slope of each calibration curve, corresponds to 48.8 ± 3.2 µA µM^−1^ cm^−2^ (*n* = 3 independent curves). The calculated limit of detection (LOD), based on the standard deviation of the response of the blank (LOD = 3.3σ_blank_/slope), corresponds to 0.065 µM. These are interesting analytical results considering an unmodified and unconventional transducing material. The CPS shows better values of LOD, and lower limit linearity and sensitivity than most studies in the literature presented in Table 1, being, therefore, a potential alternative to more conventional or expensive electrodes such as the GCE or BDD, and also to disposable screen-printed electrodes (SPE). Considering GCE-based sensors, all have employed some level of nanostructuration in order to achieve sub-micromolar LODs in trimethoprim determination, which, consequently, increases the complexity, cost, and, possibly, the environmental burden of the proposals. Despite being nanostructured, practically all these sensors present higher values for the lower limit of linearity, as well as lower sensitivities (with the exception of the sensor from Bhengo et al. [25] who achieved about five times higher sensitivity). Additionally, GCE transducers require mechanical and/or electrochemical cleaning processes to obtain reproducible results, increasing the time of analysis. Results in the same order of magnitude in terms of LOD as the one reached in this study were previously obtained by BDD electrodes [29,30] that were also used without modification with any (nano)material. However, in these sensors, the lower limit linearity were 1.7 [29] and 14 times [30] higher than the one developed in this study, also presenting a fraction of the sensitivity value (0.67 µA µM^−1^) [30] compared with the CPS. In addition, BDD is an expensive electrode material that also requires an electrochemical pre-treatment for its proper activation. Other interesting proposals using screen-printed electrodes (SPE) or carbon paste electrodes (CPE) obtained LODs in the same range, although with higher values for lower limit linearity (2 [33,35] and 20 times [32] higher) and lower sensitivity (0.03 [32,33] to 0.37 µA µM^−1^ [35]). Similarly, a hanging mercury drop electrode obtained an excellent LOD of 0.008 µM [36], although these type of electrodes are not commonly used due to the associated environmental problems of mercury. In this sense, the simplicity of the CPS predicts much lower processing costs and environmental footprints compared with the existing modified sensors (Table 1). The high sensitivity achieved by CPS can be explained by the specific nature of this transducer. It is composed of randomly arranged and tortuous carbon fibers with micrometer diameter, presenting high porosity and a format similar to paper [21,46].

The repeatability and reproducibility features of the sensor were also evaluated. In the first case, the same sensor was used to measure the same concentration of trimethoprim (0.5 µM) seven times, obtaining a relative standard deviation (RSD) of 6% for peak height (Figure S2a,b). As for the reproducibility, five different CPS applied for the same concentration of trimethoprim (0.5 µM) resulted in a RSD of about 9% for the peak height (Figure S2c,d). The reusability of CPS was not evaluated here, being discarded after a measurement, since we noted a loss in performance when reused in following days. Therefore, its assessment, as well as forms of electrode regeneration, may be pertinent future studies, accounting also for the sustainability of the CPS in the analysis of this type of analyte.

Several compounds were tested as potential interferents in the oxidation of trimethoprim (Table 2), with the interference level being expressed as the current ratio of trimethoprim peak in the absence and presence of other compounds (%current ratio = i_trimethoprim + interferent_/i_trimethoprim_ × 100). Four different and widely used pharmaceutical drugs (sulfamethoxazole, acetaminophen, amoxicillin, and aspirin) were tested individually and then in a mixture. Sulfamethoxazole, which is an antibiotic commonly prescribed with trimethoprim, shows the most significant interference, with the trimethoprim peak lowering about 14% in the presence of this drug. Other widely available compounds such as ascorbic acid, glutamic acid, glucose, lactose, sodium sulphate, and calcium carbonate were considered as interferents, though their mixture only exerted a 3% difference in trimethoprim signal (Figure S3).

### 3.4. Real Sample

The CPS was finally validated in fish samples, since aquatic species are susceptible to the bioavailability of pharmaceutical compounds released into aquatic ecosystems. The complexity of the sample requires the employment of a solid-phase extraction procedure using QuEChERS salts and dispersive kits for partitioning and cleaning of the sample from interfering compounds such as vitamins, fat, proteins, etc. Even applying this extraction procedure, the resulting signals for spiked samples were lower when compared with the analysis in buffered conditions at the same concentration level (Figure S4a), and so the determination of trimethoprim was performed by the standard addition method (Figure S4b). Analysing the fish extract directly, no trimethoprim was detected. Considering a spiking level of 0.25 µM, for three different independent analyses, the obtained recoveries vary from 103.3 to 107.4%, with RSD of 1.8% (Table 3). Through observation of Table 1, no other study has considered such complex samples in the validation of the sensors.

## 4. Conclusions

In this work, an unmodified and untreated carbon paper sensor shows good analytical results towards the determination of a potentially environmental hazardous compound, the antibiotic trimethoprim. The analytical conditions in terms of electrolyte pH, SWV parameters, and analyte electrodeposition were thoroughly optimized, with the objective to maximize the electrochemical signal and the selectivity. Ultimately, the sensor was validated in fish samples, obtaining acceptable recoveries considering the complexity of the sample nature. For instance, electrochemical sensors developed and applied to complex samples of, e.g., meat are scarce in the literature, and no specific study for trimethoprim was found. With the increase in world pollution, we can predict a contamination increase in water bodies and, consequently, aquatic species, undermining both resource sustainability and food security. Thus, a compromise of the scientific community in this field would be beneficial to create new or better determination and extraction procedures that would lead to more efficient or simple analysis for this type of sample. Additionally, the simplicity offered by the CPS due to unnecessary surface modification enables minimization of the environmental footprint of the analysis. This way, carbon paper stands as an efficient, low cost, and greener option than the more traditional electrodes, being, thus, an interesting and promising analytical tool for in situ environmental applications.

## Figures and Tables

**Table 1:** Studies in the literature concerning trimethoprim electroanalysis.

Sensor Configuration	Detection Technique	Linear Range (µM)	Sensitivity (µA µM^−1^/μA μM^−1^ cm^−2^)	LOD (µM)	Real Sample	Reference
CP	SWV	0.05–2	34.3/48.8	0.065	fish	This work
GCE/TMOPPMn(III)Cl	DPV	0.06–1	-	0.003	pharm. formul. urine	[22]
GCE/GO/PPy(MIP)	SWV	-	-	0.13	urine	[23]
GCE/rGO/AgNPs	DPV	1–10	0.1/1.41	0.4	wastewater	[24]
GCE/MWCNT/Fe_3_O_4_	DPV	0.1–0.5	162/-	0.021	pharm. formul. Water urine	[25]
GCE/CuPh-CB	SWAdASV	0.4–1.1 1.5–6	5.82/30	0.67	river water	[26]
GCE/MoO_2_	DPV	2–20	0.157/2.22	0.127	-	[27]
GCE/GR-ZnO	DPV	1–10 10–180	0.412/5.831	0.3	lake water tap water urine serum	[28]
BDD	Amperometry	0.0861–1.38	-	0.052	bovine milk	[29]
BDD	DPV	0.7–7	0.67/-	0.014	pharm. formul.	[30]
BDD	BIA–MPA	6.9–140	0.119/0.92	0.52	pharm. formul.	[31]
CPE (paraffin + MWCNT-SbNPs)	DPV	0.1–0.7	0.37/3	0.031	water	[35]
CPE/CTAB	CV	0.2–1	-	0.15	-	[34]
SPCE/rGNR	DPV	1–10	0.0303/0.433	0.04	tap water	[32]
SPE/MWCNT-PB	DPV	0.1–10	0.108/-	0.06	urine	[33]
HMDE	SW-AdCSV LS-AdCSV	0.1–1	0.45/- 0.074/-	0.01 0.008	pharm. formul.	[36]
ISE (MIP)	Potentiometry	1–1000	-	0.3	aquaculture water	[37]

AgNPs—silver nanoparticles; BDD—boron-doped diamond; CB—carbon black; CPE—carbon paste electrode; CTAB—cetyltrimethylammonium bromide; CuPh—copper (II) phthalocyanine; GCE—glassy carbon electrode; GO—graphene oxide; HMDE—hanging mercury drop electrode; ISE—ion-selective electrode; MIP—molecularly imprinted polymer; MWCNT—multi-walled carbon nanotubes; PB—Prussian blue; rGNR—reduced graphene nanoribbons; rGO—reduced graphene oxide; SbNPs—antimony nanoparticles; SPCE—screen-printed carbon electrode; SPE—screen-printed electrode; TMOPPMn(III)Cl—5,10,15,20-tetrakis(4-methoxyphenyl) porphyrinato]Mn (III)chloride.

**Table 2:** Selectivity assessment of the carbon paper sensor in the analysis of trimethoprim.

Mixture	Concentration Ratio	Interference Level (%)
Trimethoprim + sulfamethoxazole	1:1	86.0
Trimethoprim + acetaminophen	1:1	99.4
Trimethoprim + amoxicillin	1:1	106.9
Trimethoprim + aspirin	1:1	103.6
Trimethoprim + ascorbic acid + glutamic acid + glucose + lactose + sodium sulphate + calcium carbonate	1:100	97.3

**Table 3:** Recovery assays in fish samples.

Extract	Spiking (µM)	Found (µM)	Recovery (%)
1	0	n.d.	-
2	0.25	0.258	103.3
3	0.25	0.269	107.4
4	0.25	0.267	106.9
Mean		0.269	105.9
RSD		0.008	1.8

n.d.—not determined.

## Data Availability

Not applicable.

## Supplementary Materials

The following supporting information can be downloaded at: https://www.mdpi.com/article/10.3390/s23073560/s1, Figure S1: Comparison between SWV and DPV detection techniques. (a) DPV of 10 µM trimethoprim. (b) DPV of 0.1 µM trimethoprim. (c) SWV of 10 µM trimethoprim. (d) SWV of 0.1 µM trimethoprim; Figure S2: Repeatability and reproducibility of CPS sensor for 0.1 µM trimethoprim in optimized conditions. (a) Seven different SWV measurements using the same sensor to assess repeatability. (b) Comparison between peak height of the 7 measurements. (c) SWV measurements of 5 different CPS to assess reproducibility. (d) Comparison between peak height of the 5 different CPS; Figure S3: Selectivity studies performed by SWV for a trimethoprim (yellow line) concentration of 5 µM in optimized analytical conditions and for trimethoprim mixed with different compounds (ascorbic acid, glutamic acid, glucose, lactose, sodium sulphate and calcium carbonate) in a 1:500 concentration ratio (blue line); Figure S4: Example of the analysis of fish extract. (a) SWV comparing fish extract spiked with 0.25 µM of trimethoprim (full line) with 0.25 µM of trimethoprim in electrolyte solution (trace line). (b) Representative standard addition plot of the analysis of one extract spiked with 0.25 µM of trimethoprim.
