# Supplementary material for: Carbon Fiber Paper Sensor for Determination of Trimethoprim Antibiotic in Fish Samples
Source: Sensors (Basel). 2023 Mar 29;23(7):3560. doi: 10.3390/s23073560 (PMC10099197; doi:10.3390/s23073560)
Supplement: Supplementary file 1 [file sensors-23-03560-s001.zip › sensors-2290882-supplementary.pdf]

Supplementary material

Carbon fiber paper sensor for Determination of trimethoprim antibiotic in fish samples

Álvaro Torrinha, Miguel Tavares, Vitória Dibo, Cristina Delerue-Matos, Simone Morais*

REQUIMTE-LAQV, Instituto Superior de Engenharia do Porto, Instituto Politécnico do Porto, Rua Dr. António Bernardino de Almeida, 431, 4249-015 Porto, Portugal

Corresponding author:
*sbm@isep.ipp.pt (Simone Morais)

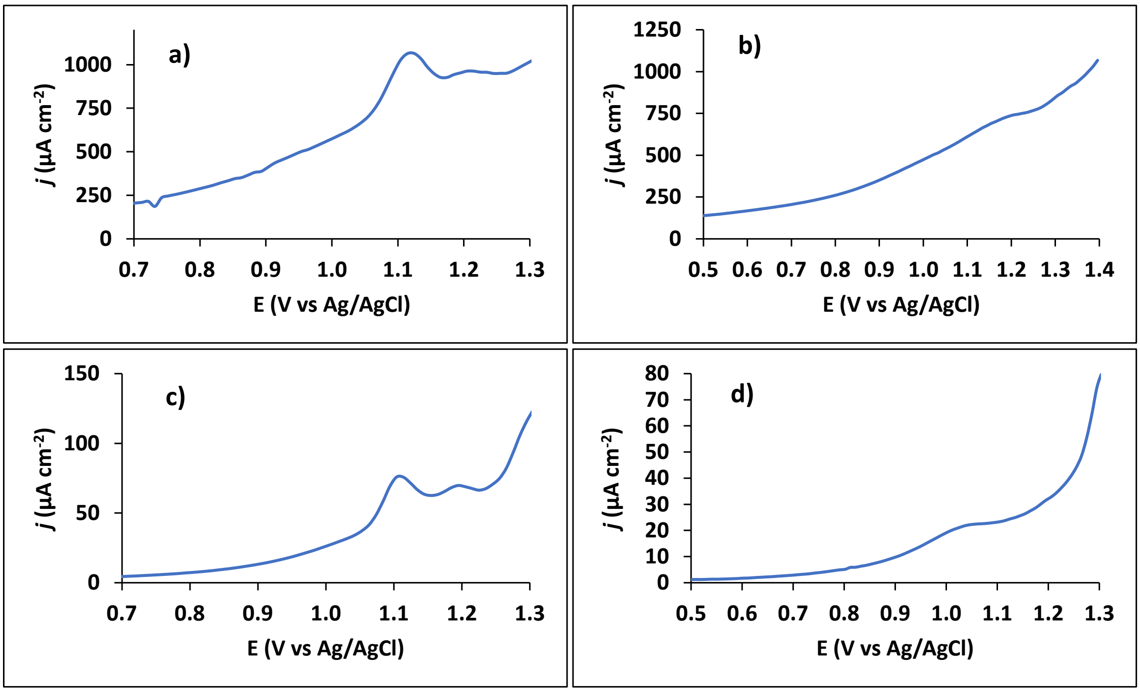

Figure S1 – Comparison between SWV and DPV detection techniques. a) DPV of 10 µM trimethoprim. b) DPV of 0.1 µM trimethoprim. c) SWV of 10 µM trimethoprim. d) SWV of 0.1 µM trimethoprim.

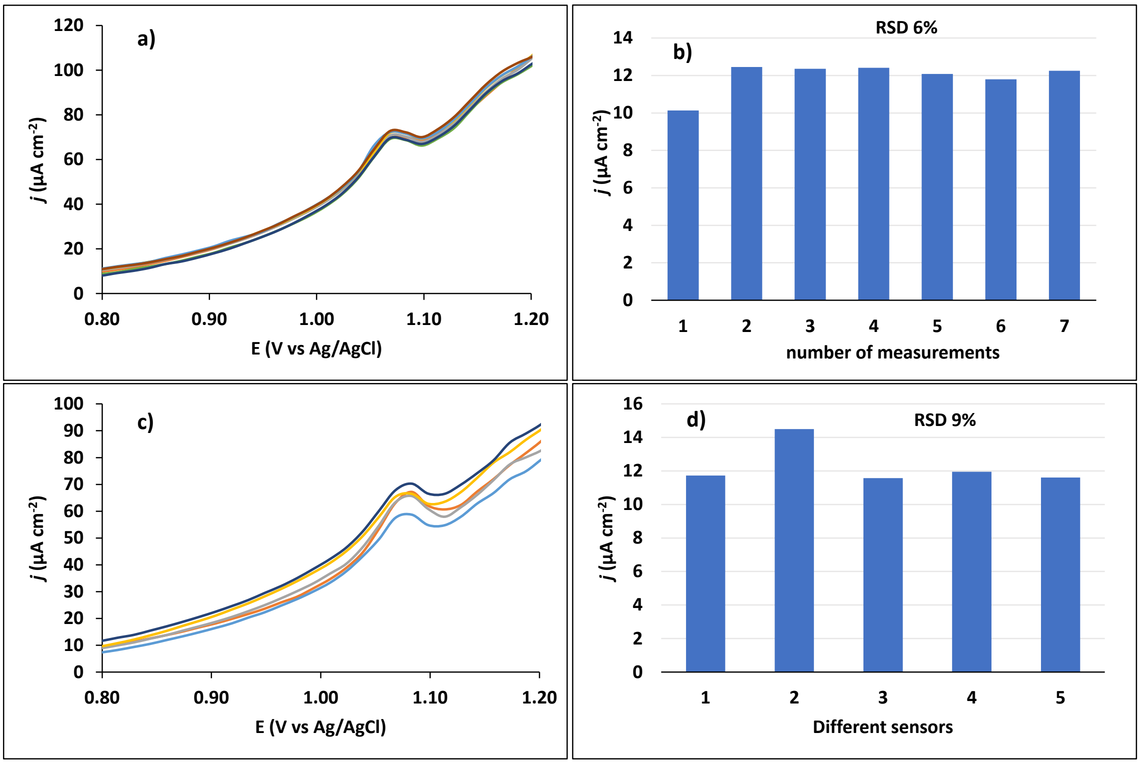

Figure S2 – Repeatability and reproducibility of CPS sensor for 0.1 µM trimethoprim in optimized conditions. a) Seven different SWV measurements using the same sensor to assess repeatability. b) Comparison between peak height of the 7 measurements. c) SWV measurements of 5 different CPS to assess reproducibility. d) Comparison between peak height of the 5 different CPS.

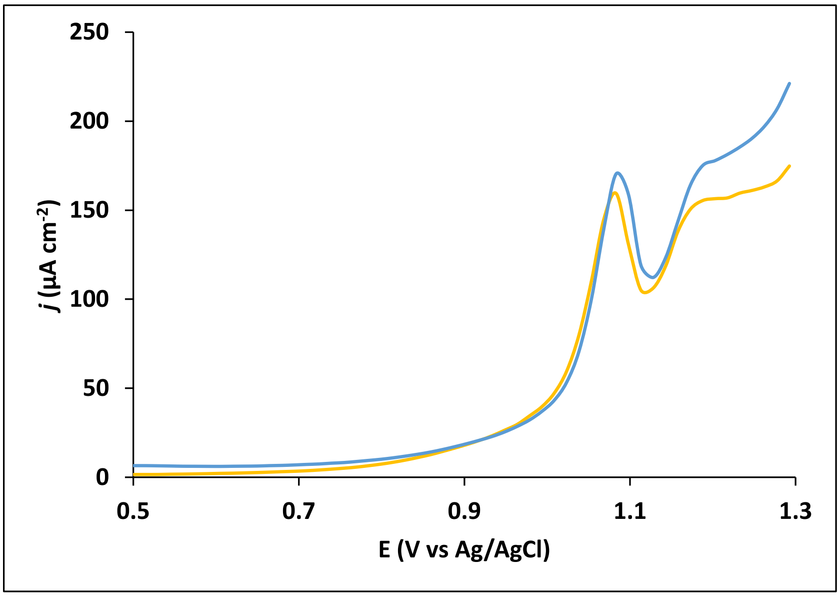

Figure S3 – Selectivity studies performed by SWV for a trimethoprim (yellow line) concentration of 5 µM in optimized analytical conditions and for trimethoprim mixed with different compounds (ascorbic acid, glutamic acid, glucose, lactose, sodium sulphate and calcium carbonate) in a 1:500 concentration ratio (blue line).

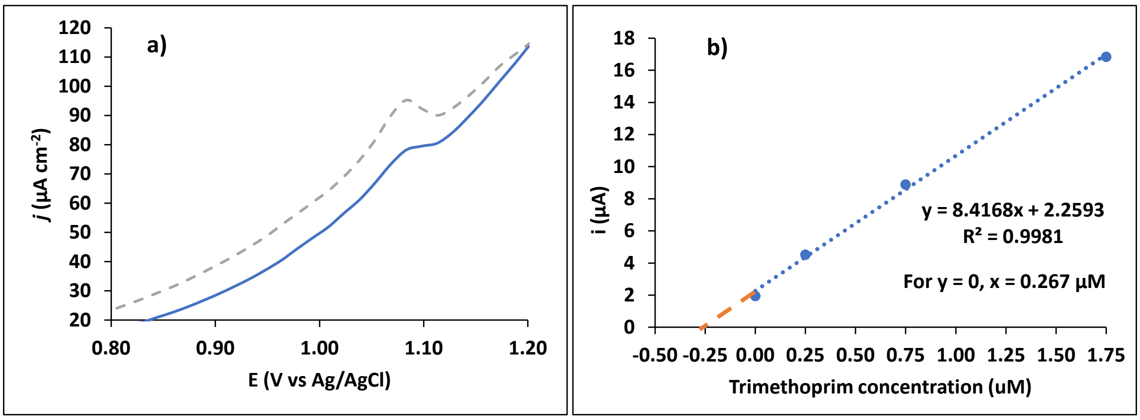

Figure S4 – Analysis of fish extract. a) SWV comparing fish extract spiked with 0.25 µM of trimethoprim (full line) with 0.25 µM of trimethoprim in electrolyte solution (trace line). b) Representative standard addition plot of the analysis of an extract spiked with 0.25 µM of trimethoprim.
